# 8-Hy­droxy-2-methyl­quinolinium diiodido(2-methyl­quinolin-8-olato-κ^2^ *N*,*O*)zincate

**DOI:** 10.1107/S1600536811032351

**Published:** 2011-08-27

**Authors:** Ezzatollah Najafi, Mostafa M. Amini, Seik Weng Ng

**Affiliations:** a Department of Chemistry, General Campus, Shahid Beheshti University, Tehran 1983963113, Iran; b Department of Chemistry, University of Malaya, 50603 Kuala Lumpur, Malaysia; c Chemistry Department, Faculty of Science, King Abdulaziz University, PO Box 80203 Jeddah, Saudi Arabia

## Abstract

The reaction of 2-methyl-8-hy­droxy­quinoline and zinc iodide in acetonitrile affords the title salt, (C_10_H_10_NO)[Zn(C_10_H_8_NO)I_2_], in which the Zn^II^ ion is coordinated by a *N*,*O*-chelating 2-methyl­quinolin-8-olate ligand and two iodide ligands in a distorted tetra­hedral geometry. The cation is linked to the anion by an O—H⋯O hydrogen bond.

## Related literature

For the crystal structures of two related 8-hy­droxy-2-methyl­quinolinium dihalo(2-methyl­quinolin-8-olato)zincate aceto­nitrile solvates, see: Najafi *et al.* (2011*a* ▶,*b* ▶). For the crystal structures of related methanol solvates, see: Najafi *et al.* (2010*a* ▶,*b* ▶); Sattarzadeh *et al.* (2009 ▶).
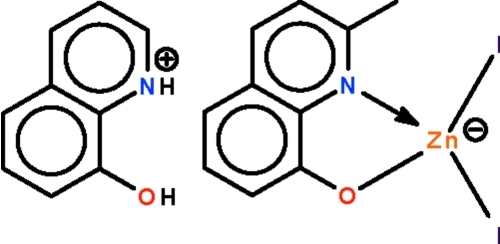


## Experimental

### 

#### Crystal data

(C_10_H_10_NO)[Zn(C_10_H_8_NO)I_2_]
*M* *_r_* = 637.53
Monoclinic, 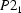

*a* = 8.1794 (2) Å
*b* = 13.9441 (3) Å
*c* = 9.1838 (2) Å
β = 102.503 (3)°
*V* = 1022.61 (4) Å^3^
*Z* = 2
Mo *K*α radiation
μ = 4.24 mm^−1^
*T* = 100 K
0.40 × 0.30 × 0.20 mm

#### Data collection

Agilent SuperNova Dual diffractometer with an Atlas detector
Absorption correction: multi-scan (*CrysAlis PRO*; Agilent, 2010 ▶) *T*_min_ = 0.282, *T*_max_ = 0.485
4995 measured reflections
3765 independent reflections
3692 reflections with *I* > 2σ(*I*)
*R*_int_ = 0.025

#### Refinement

*R*[*F* ^2^ > 2σ(*F* ^2^)] = 0.034
*wR*(*F* ^2^) = 0.087
*S* = 1.04
3765 reflections
247 parameters
1 restraint
H-atom parameters constrained
Δρ_max_ = 0.99 e Å^−3^
Δρ_min_ = −1.53 e Å^−3^
Absolute structure: Flack (1983 ▶) 1389 Friedel pairs
Flack parameter: 0.01 (2)

### 

Data collection: *CrysAlis PRO* (Agilent, 2010 ▶); cell refinement: *CrysAlis PRO*; data reduction: *CrysAlis PRO*; program(s) used to solve structure: *SHELXS97* (Sheldrick, 2008 ▶); program(s) used to refine structure: *SHELXL97* (Sheldrick, 2008 ▶); molecular graphics: *X-SEED* (Barbour, 2001 ▶); software used to prepare material for publication: *publCIF* (Westrip, 2010 ▶).

## Supplementary Material

Crystal structure: contains datablock(s) global, I. DOI: 10.1107/S1600536811032351/lh5307sup1.cif

Structure factors: contains datablock(s) I. DOI: 10.1107/S1600536811032351/lh5307Isup2.hkl

Additional supplementary materials:  crystallographic information; 3D view; checkCIF report

## Figures and Tables

**Table 1:** Hydrogen-bond geometry (Å, °)

*D*—H⋯*A*	*D*—H	H⋯*A*	*D*⋯*A*	*D*—H⋯*A*
O2—H2o⋯O1	0.84	1.71	2.542 (6)	170

**Fig. 1.**
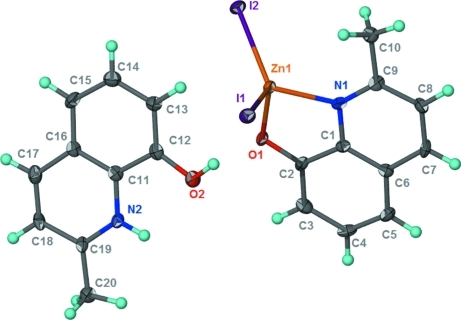
Thermal ellipsoid plot (Barbour, 2001) of (C10H10NO)[ZnI2(C10H8NO)] at the 70% probability level. Hydrogen atoms are drawn as spheres of arbitrary radius.

## supplementary crystallographic information

### Comment

We have synthesized methanol-solvated 8-hydroxy-2-methylquinolinium dihalo(2-methylquinolin-8-olato)zincates(II) by the direct reaction of the zinc halide and 8-hydroxy-2-methylquinolin in methanol. The salts have the Zn^II^ atom in a tetrahedral geometry, and the ion-pairs are linked to the solvent molecules by hydrogen bonds (Najafi *et al.*, 2010*a*; Najafi *et al.*, 2010*b*; Sattarzadeh *et al.*, 2009). These studies have been extended to the use of acetonitrile as a solvent. In a previous study, the reaction of zinc chloride/bromide and the quinoline in acetonitrile yielded the disolvated/monosolvated salts (Najafi *et al.*, 2011*a*, 2011*b*). In the present study, using zinc iodide gave a solvent-free (Fig. 1) crystal structure. In (C_10_H_10_NO)[ZnI_2_(C_10_H_8_NO)], the metal in the anion is *N*,*O*-chelated by the deprotonated ligand and it exists in a distorted tetrahedral geometry. The cation is linked to the anion by an O–H···O hydrogen bond (Table 1).

### Experimental

Zinc iodide (0.32 g, 0.75 mmol) and 2-methyl-8-hydroxyquinoline (0.24 g, 1.5 mmol) were loaded into a convection tube and the tube was filled with acetonitrile and kept at 333 K. Yellow crystals were collected from the side arm after several days.

### Refinement

Carbon-bound H-atoms were placed in calculated positions [C—H 0.95 to 0.98 Å, *U*_iso_(H) 1.2 to 1.5*U*_eq_(C)] and were included in the refinement in the riding model approximation. The N and O bound H atoms were similarly treated [N–H 0.88, O–H 0.84 Å; *U*_iso_(H) = 1.2*U*_eq_(N) or 1.5*U*_eq_(O) ]. The (-2 8 1), (-2 3 5), (-2 2 5), (-2 4 5) and (-2 5 5) reflections were removed.

### Crystal data

**Table:**

(C_10_H_10_NO)[Zn(C_10_H_8_NO)I_2_]	*F*(000) = 608
*M**_r_* = 637.53	*D*_x_ = 2.070 Mg m^−^^3^
Monoclinic, *P*2_1_	Mo *K*α radiation, λ = 0.71073 Å
Hall symbol: P 2yb	Cell parameters from 4585 reflections
*a* = 8.1794 (2) Å	θ = 2.6–26.3°
*b* = 13.9441 (3) Å	µ = 4.24 mm^−^^1^
*c* = 9.1838 (2) Å	*T* = 100 K
β = 102.503 (3)°	Block, yellow
*V* = 1022.61 (4) Å^3^	0.40 × 0.30 × 0.20 mm
*Z* = 2	

### Data collection

**Table:**

Agilent SuperNova Dual diffractometer with an Atlas detector	3765 independent reflections
Radiation source: SuperNova (Mo) X-ray Source	3692 reflections with *I* > 2σ(*I*)
Mirror	*R*_int_ = 0.025
Detector resolution: 10.4041 pixels mm^-1^	θ_max_ = 27.5°, θ_min_ = 2.6°
ω scans	*h* = −10→10
Absorption correction: multi-scan (*CrysAlis PRO*; Agilent, 2010)	*k* = −17→18
*T*_min_ = 0.282, *T*_max_ = 0.485	*l* = −11→7
4995 measured reflections	

### Refinement

**Table:**

Refinement on *F*^2^	Secondary atom site location: difference Fourier map
Least-squares matrix: full	Hydrogen site location: inferred from neighbouring sites
*R*[*F*^2^ > 2σ(*F*^2^)] = 0.034	H-atom parameters constrained
*wR*(*F*^2^) = 0.087	*w* = 1/[σ^2^(*F*_o_^2^) + (0.0676*P*)^2^ + 0.3294*P*] where *P* = (*F*_o_^2^ + 2*F*_c_^2^)/3
*S* = 1.04	(Δ/σ)_max_ = 0.001
3765 reflections	Δρ_max_ = 0.99 e Å^−^^3^
247 parameters	Δρ_min_ = −1.53 e Å^−^^3^
1 restraint	Absolute structure: Flack (1983) 1389 Friedel pairs
Primary atom site location: structure-invariant direct methods	Flack parameter: 0.01 (2)

### Fractional atomic coordinates and isotropic or equivalent isotropic displacement parameters (Å^2^)

**Table:**

	*x*	*y*	*z*	*U*_iso_*/*U*_eq_	
I1	0.34742 (4)	0.50002 (2)	0.27609 (4)	0.01513 (10)	
I2	0.86547 (4)	0.57723 (3)	0.30050 (4)	0.01526 (10)	
Zn1	0.65211 (7)	0.50245 (5)	0.42920 (6)	0.01153 (14)	
O1	0.7152 (5)	0.3768 (3)	0.5317 (4)	0.0140 (8)	
O2	0.7800 (6)	0.2102 (3)	0.4468 (5)	0.0185 (9)	
H2O	0.7556	0.2669	0.4645	0.028*	
N1	0.6329 (6)	0.5443 (4)	0.6384 (6)	0.0128 (10)	
N2	0.8374 (6)	0.0372 (4)	0.3439 (6)	0.0124 (10)	
H2N	0.7921	0.0452	0.4217	0.015*	
C1	0.6269 (7)	0.4639 (5)	0.7234 (7)	0.0118 (12)	
C2	0.6735 (7)	0.3762 (5)	0.6652 (6)	0.0127 (11)	
C3	0.6735 (8)	0.2940 (5)	0.7488 (7)	0.0164 (12)	
H3	0.7045	0.2344	0.7126	0.020*	
C4	0.6277 (7)	0.2983 (5)	0.8879 (7)	0.0180 (12)	
H4	0.6277	0.2407	0.9431	0.022*	
C5	0.5831 (8)	0.3824 (5)	0.9472 (7)	0.0176 (12)	
H5	0.5521	0.3831	1.0411	0.021*	
C6	0.5844 (7)	0.4686 (5)	0.8641 (7)	0.0134 (12)	
C7	0.5476 (7)	0.5605 (5)	0.9138 (6)	0.0163 (14)	
H7	0.5153	0.5670	1.0067	0.020*	
C8	0.5583 (8)	0.6396 (5)	0.8297 (7)	0.0156 (12)	
H8	0.5373	0.7014	0.8654	0.019*	
C9	0.6008 (8)	0.6297 (5)	0.6886 (7)	0.0134 (13)	
C10	0.6195 (8)	0.7153 (4)	0.5938 (7)	0.0160 (12)	
H10A	0.5744	0.7723	0.6337	0.024*	
H10B	0.5580	0.7040	0.4912	0.024*	
H10C	0.7383	0.7254	0.5947	0.024*	
C11	0.8860 (8)	0.1179 (5)	0.2762 (7)	0.0137 (12)	
C12	0.8575 (8)	0.2094 (5)	0.3348 (8)	0.0144 (12)	
C13	0.9136 (8)	0.2891 (4)	0.2680 (7)	0.0164 (12)	
H13	0.8988	0.3516	0.3043	0.020*	
C14	0.9923 (8)	0.2774 (5)	0.1470 (7)	0.0190 (13)	
H14	1.0315	0.3328	0.1049	0.023*	
C15	1.0147 (8)	0.1899 (5)	0.0878 (7)	0.0179 (12)	
H15	1.0655	0.1847	0.0044	0.021*	
C16	0.9607 (8)	0.1066 (4)	0.1530 (7)	0.0133 (12)	
C17	0.9785 (8)	0.0115 (5)	0.1023 (7)	0.0172 (12)	
H17	1.0273	0.0012	0.0187	0.021*	
C18	0.9261 (8)	−0.0647 (4)	0.1728 (7)	0.0156 (12)	
H18	0.9374	−0.1278	0.1372	0.019*	
C19	0.8552 (7)	−0.0510 (6)	0.2982 (7)	0.0130 (15)	
C20	0.8021 (8)	−0.1330 (5)	0.3803 (7)	0.0187 (12)	
H20A	0.7757	−0.1098	0.4733	0.028*	
H20B	0.8930	−0.1800	0.4033	0.028*	
H20C	0.7027	−0.1631	0.3186	0.028*	

### Atomic displacement parameters (Å^2^)

**Table:**

	*U*^11^	*U*^22^	*U*^33^	*U*^12^	*U*^13^	*U*^23^
I1	0.01258 (17)	0.01704 (18)	0.01573 (17)	−0.00109 (15)	0.00297 (12)	0.00069 (15)
I2	0.01426 (17)	0.01766 (18)	0.01540 (17)	−0.00195 (15)	0.00660 (12)	0.00132 (15)
Zn1	0.0134 (3)	0.0116 (3)	0.0105 (3)	0.0006 (3)	0.0048 (2)	0.0002 (3)
O1	0.021 (2)	0.0107 (18)	0.012 (2)	0.0033 (17)	0.0093 (16)	0.0002 (16)
O2	0.025 (2)	0.0118 (19)	0.022 (2)	0.0044 (18)	0.0115 (18)	−0.0004 (17)
N1	0.012 (2)	0.014 (2)	0.013 (2)	−0.001 (2)	0.0035 (18)	0.003 (2)
N2	0.013 (2)	0.013 (2)	0.012 (2)	0.003 (2)	0.0051 (19)	−0.001 (2)
C1	0.010 (3)	0.013 (3)	0.012 (3)	−0.001 (2)	0.002 (2)	0.001 (2)
C2	0.011 (3)	0.017 (3)	0.010 (3)	−0.002 (2)	0.003 (2)	0.000 (2)
C3	0.017 (3)	0.011 (3)	0.020 (3)	0.002 (2)	0.003 (2)	0.000 (2)
C4	0.019 (3)	0.016 (3)	0.019 (3)	−0.001 (3)	0.006 (2)	0.008 (2)
C5	0.021 (3)	0.021 (3)	0.013 (3)	−0.005 (3)	0.007 (2)	0.000 (2)
C6	0.012 (3)	0.013 (3)	0.015 (3)	−0.001 (2)	0.002 (2)	0.000 (2)
C7	0.020 (3)	0.022 (4)	0.008 (3)	0.001 (3)	0.005 (2)	−0.003 (2)
C8	0.018 (3)	0.012 (3)	0.017 (3)	−0.002 (2)	0.004 (2)	−0.004 (2)
C9	0.012 (3)	0.012 (3)	0.016 (3)	0.007 (3)	0.004 (2)	0.003 (2)
C10	0.017 (3)	0.013 (3)	0.018 (3)	0.003 (2)	0.004 (2)	0.001 (2)
C11	0.012 (3)	0.013 (3)	0.016 (3)	0.003 (2)	0.002 (2)	0.000 (2)
C12	0.012 (3)	0.015 (3)	0.014 (3)	0.001 (2)	−0.001 (2)	−0.002 (2)
C13	0.019 (3)	0.013 (3)	0.017 (3)	−0.001 (2)	0.005 (2)	0.001 (2)
C14	0.021 (3)	0.018 (3)	0.019 (3)	−0.002 (2)	0.004 (2)	0.001 (2)
C15	0.018 (3)	0.023 (3)	0.014 (3)	−0.001 (3)	0.008 (2)	0.001 (2)
C16	0.011 (2)	0.013 (3)	0.015 (3)	0.003 (2)	0.002 (2)	−0.001 (2)
C17	0.014 (3)	0.020 (3)	0.018 (3)	0.003 (3)	0.005 (2)	−0.006 (3)
C18	0.019 (3)	0.011 (3)	0.016 (3)	0.006 (2)	0.005 (2)	−0.004 (2)
C19	0.016 (3)	0.013 (3)	0.010 (3)	0.003 (2)	0.002 (2)	−0.001 (2)
C20	0.022 (3)	0.016 (3)	0.019 (3)	−0.001 (2)	0.006 (2)	−0.001 (2)

### Geometric parameters (Å, °)

**Table:**

I1—Zn1	2.5831 (7)	C8—C9	1.420 (9)
I2—Zn1	2.5343 (7)	C8—H8	0.9500
Zn1—O1	2.003 (4)	C9—C10	1.505 (9)
Zn1—N1	2.046 (5)	C10—H10A	0.9800
O1—C2	1.342 (7)	C10—H10B	0.9800
O2—C12	1.320 (8)	C10—H10C	0.9800
O2—H2O	0.8400	C11—C16	1.406 (9)
N1—C9	1.323 (8)	C11—C12	1.423 (9)
N1—C1	1.373 (8)	C12—C13	1.394 (9)
N2—C19	1.317 (9)	C13—C14	1.409 (8)
N2—C11	1.386 (8)	C13—H13	0.9500
N2—H2N	0.8800	C14—C15	1.365 (9)
C1—C6	1.410 (9)	C14—H14	0.9500
C1—C2	1.420 (9)	C15—C16	1.420 (9)
C2—C3	1.380 (9)	C15—H15	0.9500
C3—C4	1.407 (8)	C16—C17	1.423 (9)
C3—H3	0.9500	C17—C18	1.362 (9)
C4—C5	1.376 (9)	C17—H17	0.9500
C4—H4	0.9500	C18—C19	1.409 (8)
C5—C6	1.424 (9)	C18—H18	0.9500
C5—H5	0.9500	C19—C20	1.486 (9)
C6—C7	1.415 (9)	C20—H20A	0.9800
C7—C8	1.360 (9)	C20—H20B	0.9800
C7—H7	0.9500	C20—H20C	0.9800
			
O1—Zn1—N1	82.69 (19)	C8—C9—C10	121.8 (6)
O1—Zn1—I2	116.46 (12)	C9—C10—H10A	109.5
N1—Zn1—I2	121.41 (14)	C9—C10—H10B	109.5
O1—Zn1—I1	111.38 (13)	H10A—C10—H10B	109.5
N1—Zn1—I1	104.68 (14)	C9—C10—H10C	109.5
I2—Zn1—I1	115.60 (3)	H10A—C10—H10C	109.5
C2—O1—Zn1	110.1 (3)	H10B—C10—H10C	109.5
C12—O2—H2O	109.5	N2—C11—C16	119.1 (6)
C9—N1—C1	120.2 (5)	N2—C11—C12	118.2 (6)
C9—N1—Zn1	130.2 (4)	C16—C11—C12	122.7 (6)
C1—N1—Zn1	108.7 (4)	O2—C12—C13	126.5 (6)
C19—N2—C11	123.5 (5)	O2—C12—C11	116.6 (6)
C19—N2—H2N	118.2	C13—C12—C11	116.8 (6)
C11—N2—H2N	118.2	C12—C13—C14	120.3 (6)
N1—C1—C6	122.0 (6)	C12—C13—H13	119.8
N1—C1—C2	116.4 (5)	C14—C13—H13	119.8
C6—C1—C2	121.6 (6)	C15—C14—C13	122.7 (6)
O1—C2—C3	123.0 (6)	C15—C14—H14	118.6
O1—C2—C1	118.7 (5)	C13—C14—H14	118.6
C3—C2—C1	118.3 (5)	C14—C15—C16	118.9 (5)
C2—C3—C4	120.1 (6)	C14—C15—H15	120.6
C2—C3—H3	119.9	C16—C15—H15	120.6
C4—C3—H3	119.9	C11—C16—C15	118.4 (6)
C5—C4—C3	122.8 (5)	C11—C16—C17	117.5 (6)
C5—C4—H4	118.6	C15—C16—C17	124.1 (5)
C3—C4—H4	118.6	C18—C17—C16	120.4 (5)
C4—C5—C6	118.3 (5)	C18—C17—H17	119.8
C4—C5—H5	120.9	C16—C17—H17	119.8
C6—C5—H5	120.9	C17—C18—C19	120.7 (6)
C1—C6—C7	116.7 (6)	C17—C18—H18	119.7
C1—C6—C5	119.0 (6)	C19—C18—H18	119.7
C7—C6—C5	124.3 (5)	N2—C19—C18	118.8 (6)
C8—C7—C6	120.5 (5)	N2—C19—C20	119.4 (5)
C8—C7—H7	119.8	C18—C19—C20	121.8 (6)
C6—C7—H7	119.8	C19—C20—H20A	109.5
C7—C8—C9	119.9 (6)	C19—C20—H20B	109.5
C7—C8—H8	120.1	H20A—C20—H20B	109.5
C9—C8—H8	120.1	C19—C20—H20C	109.5
N1—C9—C8	120.8 (6)	H20A—C20—H20C	109.5
N1—C9—C10	117.3 (5)	H20B—C20—H20C	109.5
			
N1—Zn1—O1—C2	15.2 (4)	C6—C7—C8—C9	2.2 (9)
I2—Zn1—O1—C2	136.8 (3)	C1—N1—C9—C8	−0.7 (9)
I1—Zn1—O1—C2	−87.7 (4)	Zn1—N1—C9—C8	166.6 (4)
O1—Zn1—N1—C9	175.6 (6)	C1—N1—C9—C10	176.5 (5)
I2—Zn1—N1—C9	59.0 (6)	Zn1—N1—C9—C10	−16.3 (9)
I1—Zn1—N1—C9	−74.1 (6)	C7—C8—C9—N1	−1.0 (9)
O1—Zn1—N1—C1	−15.9 (4)	C7—C8—C9—C10	−178.0 (6)
I2—Zn1—N1—C1	−132.6 (3)	C19—N2—C11—C16	−0.8 (8)
I1—Zn1—N1—C1	94.3 (4)	C19—N2—C11—C12	179.0 (6)
C9—N1—C1—C6	1.1 (9)	N2—C11—C12—O2	−2.6 (8)
Zn1—N1—C1—C6	−168.6 (5)	C16—C11—C12—O2	177.2 (5)
C9—N1—C1—C2	−176.1 (6)	N2—C11—C12—C13	177.6 (5)
Zn1—N1—C1—C2	14.1 (6)	C16—C11—C12—C13	−2.6 (9)
Zn1—O1—C2—C3	167.8 (5)	O2—C12—C13—C14	−178.9 (6)
Zn1—O1—C2—C1	−12.1 (6)	C11—C12—C13—C14	0.9 (9)
N1—C1—C2—O1	−1.7 (8)	C12—C13—C14—C15	1.3 (10)
C6—C1—C2—O1	−178.9 (6)	C13—C14—C15—C16	−1.7 (9)
N1—C1—C2—C3	178.3 (5)	N2—C11—C16—C15	−178.0 (5)
C6—C1—C2—C3	1.1 (9)	C12—C11—C16—C15	2.2 (9)
O1—C2—C3—C4	−179.8 (5)	N2—C11—C16—C17	1.4 (8)
C1—C2—C3—C4	0.2 (9)	C12—C11—C16—C17	−178.4 (6)
C2—C3—C4—C5	−0.6 (10)	C14—C15—C16—C11	0.0 (9)
C3—C4—C5—C6	−0.3 (9)	C14—C15—C16—C17	−179.4 (6)
N1—C1—C6—C7	0.1 (9)	C11—C16—C17—C18	−0.6 (9)
C2—C1—C6—C7	177.1 (5)	C15—C16—C17—C18	178.8 (6)
N1—C1—C6—C5	−179.1 (5)	C16—C17—C18—C19	−0.8 (9)
C2—C1—C6—C5	−2.0 (9)	C11—N2—C19—C18	−0.6 (9)
C4—C5—C6—C1	1.6 (9)	C11—N2—C19—C20	178.8 (6)
C4—C5—C6—C7	−177.5 (6)	C17—C18—C19—N2	1.4 (9)
C1—C6—C7—C8	−1.7 (9)	C17—C18—C19—C20	−178.0 (6)
C5—C6—C7—C8	177.4 (6)		

### Hydrogen-bond geometry (Å, °)

**Table:**

*D*—H···*A*	*D*—H	H···*A*	*D*···*A*	*D*—H···*A*
O2—H2o···O1	0.84	1.71	2.542 (6)	170
